# A Rare Combination of Heptadactyl and Hexadactyl Polydactyly in a Neonate

**DOI:** 10.7759/cureus.37920

**Published:** 2023-04-21

**Authors:** Jagdish Mujalda, Anshu Mujalda, Deepak Reddy, Sanjay Rai, Himakshi Modi

**Affiliations:** 1 Paediatrics, Military Hospital Ambala, Ambala, IND; 2 Obstetrics and Gynaecology, Maharishi Markandeshwar Institute of Medical Sciences and Research (MMIMSR), Multana, IND; 3 Radiology, Military Hospital Ambala, Ambala, IND; 4 Orthopaedics, 151 Base Hospital, Guwahati, IND

**Keywords:** neonate, congenital, supernumerary toe, polydactyly hand, hexadactylia foot

## Abstract

Heptadactyly and hexadactyly are rare congenital disorders from the polydactyly family. This type of polydactyly is usually classified into three major groups: preaxial (medial ray), postaxial (lateral ray), and central polydactyly. The most common presentation is both preaxial and postaxial polydactyly. The occurrence of heptadactyly and hexadactyly has been reported but the presence of both in the same infant has not been reported yet. We report the presence of both these abnormalities in the same infant.

## Introduction

Polydactyly, also called hyperdactyly is a common congenital limb abnormality characterized by extra toes or fingers. It is invariably associated with phenotypes (syndromic polydactyly) or may present as a separate entity (non-syndromic polydactyly) [1,2]. It can present in isolation or as a part of a syndromic disease [3,4]. When it occurs in isolation, it is typically inherited in an autosomal dominant fashion [5]. On the other hand, it usually tends to be autosomal recessive when it is associated with other diseases and syndromes [6]. Some of the associated syndromes include Holt-Oram syndrome, Down’s syndrome, polycythemia, Meckel syndrome, Laurence-Moon-Bardet-Biedl syndrome, Patau’s syndrome, and Klippel-Trenaunay syndrome [3].

## Case presentation

The patient is a newborn male, born to a G5P2L2A2 (gravida 5, para 2, live baby 2, abortion 2) mother. The baby was delivered by repeat lower segment caesarian section (LSCS) to non-consanguineous parents. The patient had two elder sisters who were medically fit with no history of polydactyly. We did not find any family history of polydactyly or any genetic disorders. 

**Figure 1:**
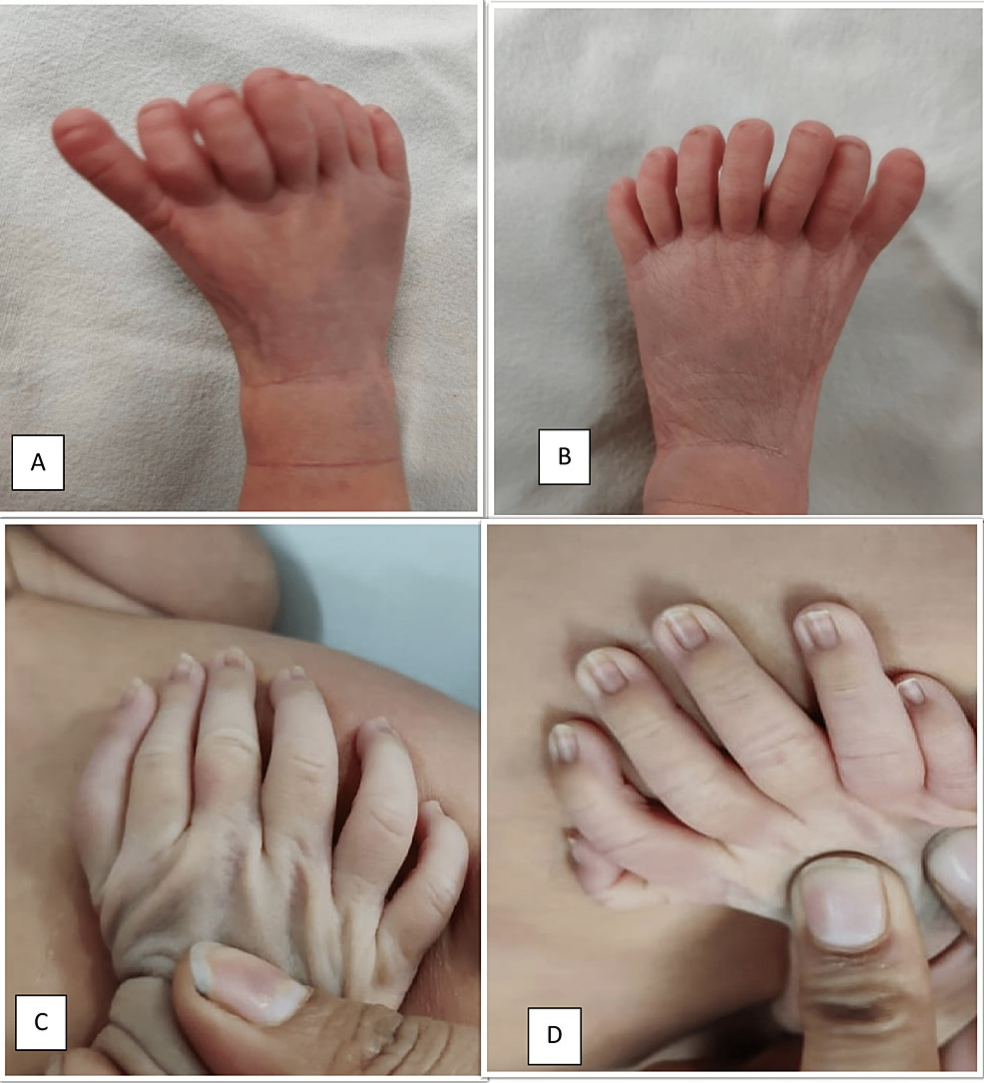
Distribution of polydactyly in feet and hands showing: (A) seven toes in right foot; (B) seven toes in left foot; (C) six fingers in left hand; (D) six fingers in right hand Written consent from parents has been taken to publish their baby's pictures for medical education purposes.

**Figure 2:**
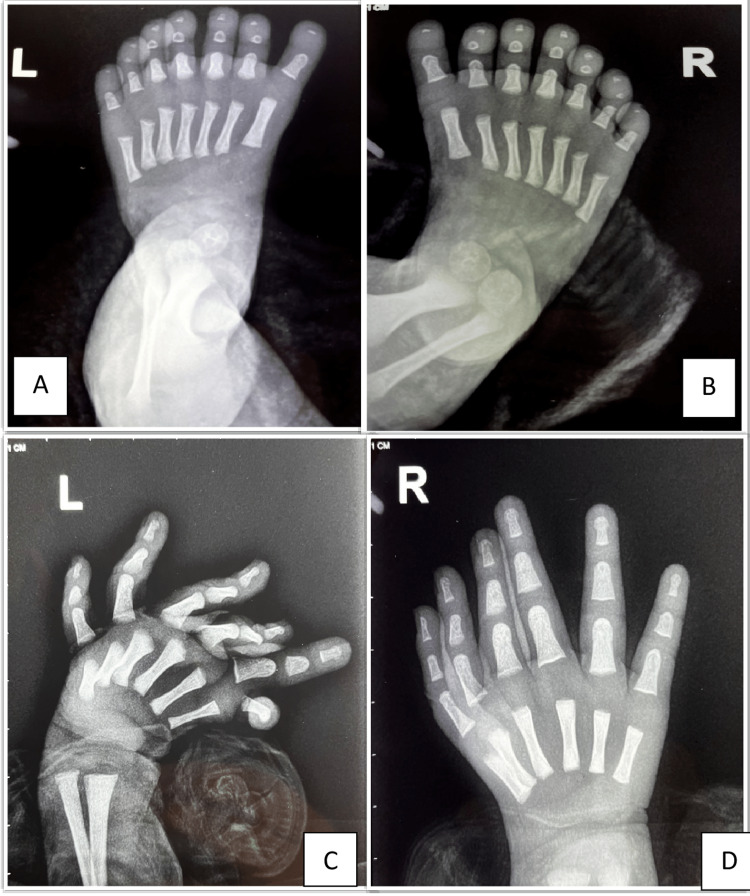
X-ray of both hands and feet showing distribution and number of digits Written consent from parents has been taken to publish their baby's pictures for medical education purposes.

**Figure 3:**
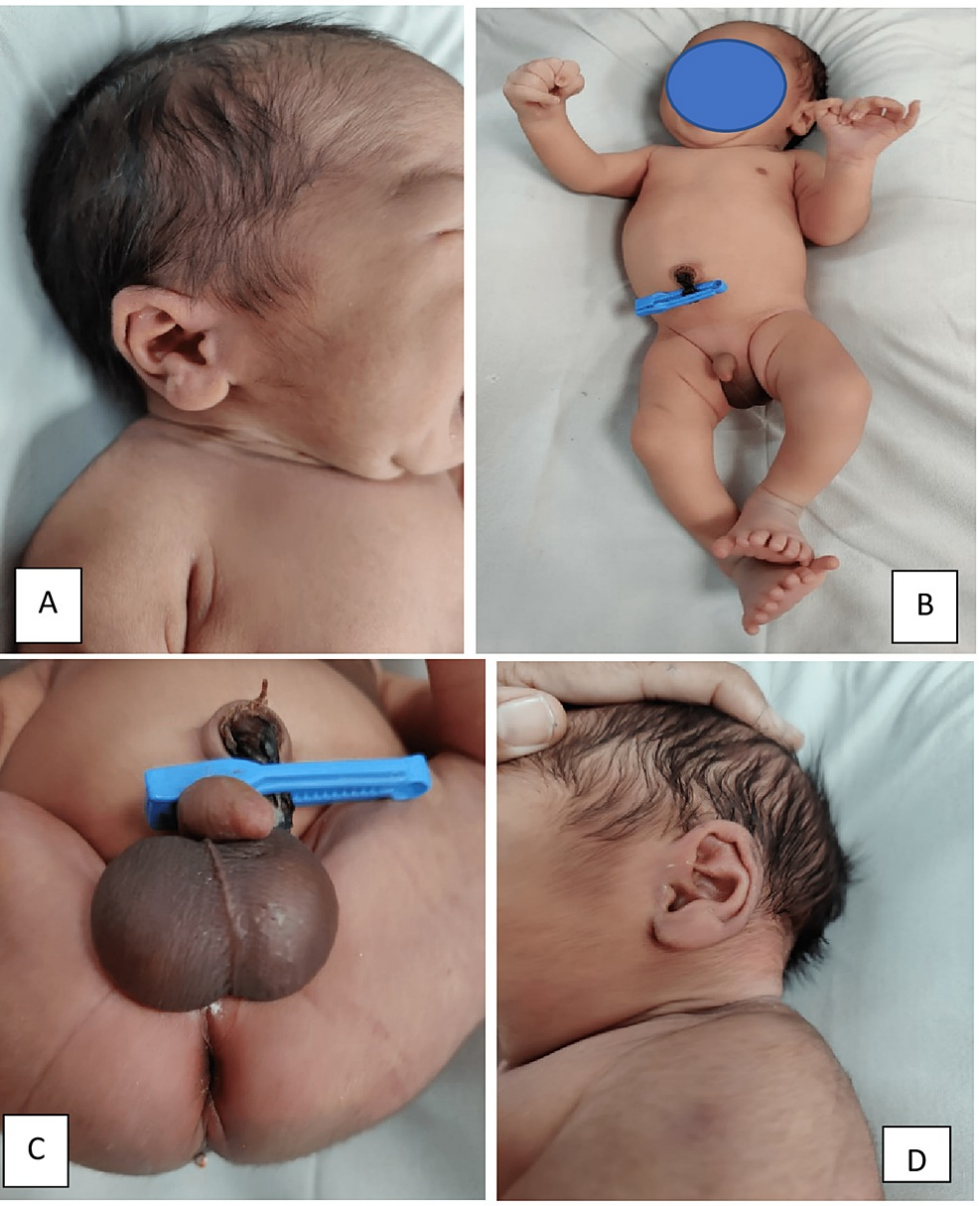
Full clinical photographs of infant showing: (A) normal appearance of right side of neck and pinna; (B) the whole body; (C) genitalia with normal appearance; (D) normal appearance of left side of neck and pinna Written consent from parents has been taken to publish their baby's pictures for medical education purposes.

Antenatal history comprised a spontaneous conception with full anti-natal workup and ultrasonography as per protocol. There was no history of vomiting, fever, rash, headache, burning micturition, drug intake, or any radiation exposure. Head-to-toe examination revealed an extra digit in each hand and two extra digits in each foot (Figures 1, 2, 3).

X-ray of the hands and feet showed extra full rays in both feet and hands, which indicates fully developed and functional rays, (Figure 2). Ultrasonography of the abdomen reveals no intra-abdominal organ anomaly. Similarly, echocardiography showed normal heart structure and function.

Full body clinical photographs show the distribution of polydactyly in feet and hands. The neck size, pinna, genitalia, and hairline, seem to be normal (Figure 3).

**Figure 4:**
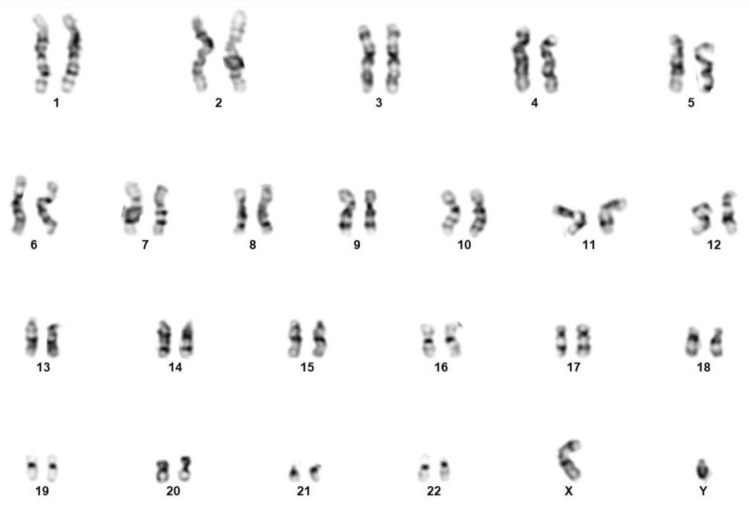
Karyotyping analysis, which does not reveal any abnormity

It is important to perform karyotyping of parents and the neonate in such cases. In the present case, karyotyping analyses of the parents and the infant were normal (Figure 4).

His laboratory values were as follows: white blood cell (WBC), 18000/cu mm; C-reactive protein (CRP), 14.8 mg/dl; aspartate aminotransferase (AST), 38 U/L; alanine transaminase (ALT), 72 U/L; alkaline phosphatase (ALP), 180 U/L; gamma-glutamyl transferase (GGT), 105 U/L; total bilirubin, 1.3 mg/dl; and unconjugated bilirubin, 0.72 mg/dl (Table 1).

**Table 1:** Haematological investigations SGPT: serum glutamate pyruvate transaminase

Parameters	Values
Haemoglobin	10.4 g/dL
Total Leukocyte count	18000 /cu mm
Urea	16.3 mg/dL
Sugar	98 mg/dL
Total serum bilirubin	1.3 mg/dL
Serum direct bilirubin	0.49 mg/dL
Total Proteins	4.67 mg/dL
SGPT	43.5U/L
Platelets	670044/ cu mm
C-reactive protein (CRP)	14.8 mg/dl
Alkaline phosphatase (ALP)	180 U/L
Aspartate aminotransferase (AST)	38 U/L
Gamma-glutamyl transferase (GGT)	105 U/L

Institutional Ethical Committee has been taken (approval number: MHA/Pedia/01/2023). Written consent from parents has been taken to publish their baby's photographs for medical education purposes.

## Discussion

Polydactyly or polydactylism, also called hyperdactyly, is a congenital limb abnormality with varied morphologic phenotypes. In addition to physical and functional impairments, its presence may be an indication of underlying intra-abdominal, thoracic, or cardiac anomalies and other associated syndromes. Polydactyly follows an autosomal dominant/recessive inheritance pattern. Polydactyly is a multifactorial, multigenetic disorder as many genes have been found to play a role in its pathogenesis [7].

Polydactyly can present on the radial/tibial side (preaxial), ulnar/fibular side (postaxial), or involve non-border central digits [8]. Watt and Chung have reported the incidence of preaxial polydactyly (PPD) as high as one in 3000 births [9]. Zun et al. have reported a polydactyly incidence of one per 1,000 live births [10].

Deng et al. [11] and, Manske et al. [12] reported that in PPD, the extra digit is located near the first digit of the hand (radial side; thumb) or foot (medially). They also reported the incidence of PPD as 0.8 to 2.3 in 10,000 live births.

PPD is generally observed as an isolated anomaly, of which spontaneous mutations might be the possible cause [13]. PPD occurs due to malfunction of ectodermal and preaxial mesodermal apoptosis in the early developmental stage of an embryo before the eighth week of gestation. Bouldin and Harfe showed that an abnormal expression of genes like Hox genes, hedgehog genes, bone morphogenic proteins (BMPs), and GLI3 may be the cause of the development of PPD [14]. According to Wassel, thumb polydactylia also exist in seven different types based on skeletal duplication. Starting from distal to proximal, types 1, 3, and 5 refer to bifid phalanges, and types 2, 4, and 6 refer to complete phalangeal duplication [15].

Polydactyly involving both hands and feet is rare [16]. In this report, we describe a case of tetrapolydactyly (26 digits) in a male neonate. Polydactyly is the most common congenital hand and foot anomaly seen in infants [1]. However, tetrapolydactyly is very rare among polydactyly and is more common in male infants as compared to female infants [12]. In the present report, the infant is a male born to Indian parents with no family history of such congenital abnormalities in the family.

As far as the functionality of the extra digit is concerned, it may be partially functional or non-functional due to a lack of muscular connections [17]. Rayan and Frey reported a fleshy nubbin as a form of classification [18]. In the present case, however, we report fully functional extra digits in both hands and feet with intact voluntary movements.

## Conclusions

Advances in human genetics have shown various isolated and syndromic polydactyly types that enable us to understand the genes responsible for limb anomalies. The genetic pathway of polydactyly is complex and has not yet been completely understood, and not merely restricted to Mendelian inheritance. Factors like associated genes, genetic and allelic heterogeneity, enhancers/suppressors, and various environmental and developmental factors play a vital role. 

Tetrapolydactyly is a rare congenital anomaly worldwide, and in this report, we present its occurrence in a male infant without associated congenital anomalies. We recommend performing a detailed clinical, radiological, and ultrasonographical examination to exclude other concomitant congenital abnormalities. Its management requires a multi-specialist team which includes an orthopaedics surgeon, reconstruction surgeon, and vascular surgeon. 
